# In Vitro and In Silico Protocols for the Assessment of Anti-Tick Compounds from *Pinus roxburghii* against *Rhipicephalus (Boophilus) microplus* Ticks

**DOI:** 10.3390/ani13081388

**Published:** 2023-04-18

**Authors:** Sana Ayub, Nosheen Malak, Raquel Cossío-Bayúgar, Nasreen Nasreen, Afshan Khan, Sadaf Niaz, Adil Khan, Abdallah D. Alanazi, Mourad Ben Said

**Affiliations:** 1 Department of Zoology, Abdul Wali Khan University Mardan, Mardan 23200, Pakistan; 2 Centro Nacional de Investigaciones Disciplinarias en Salud Animal e Inocuidad, Departamento de Artropodología, Instituto Nacional de Investigaciones Forestales Agrícolas y Pecuarias (INIFAP), Boulevard Cuauhnahuac No. 8534, Jiutepec 62574, Mexico; 3 Department of Zoology, Bacha Khan University Charsadda, Charsadda 24420, Pakistan; 4 Department of Biological Sciences, Faculty of Science and Humanities, Shaqra University, Ad-Dawadimi 11911, Saudi Arabia; 5 Department of Basic Sciences, Higher Institute of Biotechnology of Sidi Thabet, University of Manouba, Manouba 2010, Tunisia; 6 Laboratory of Microbiology, National School of Veterinary Medicine, Sidi Thabet, University of Manouba, Manouba 2010, Tunisia

**Keywords:** *Rhipicephalus (Boophilus) microplus* ticks, acaricidal activity, *Pinus roxburghii*, cypermethrin, plant extract, molecular docking

## Abstract

**Simple Summary:**

*Rhipicephalus* (*Boophilus*) *microplus*, commonly known as the cattle tick, is an ectoparasite that affects all terrestrial vertebrates, including livestock. As animal husbandry is the backbone of Pakistan’s economy, tick infestation results in significant economic losses for farmers annually. Given the reported resistance of various tick species to chemical acaricides, a recent study investigated the effectiveness of *Pinus roxburghii* plant extract in controlling tick populations. This study observed that the plant extract showed high efficacy in vitro against different tick stages in adult immersion test and larval packet test. Additionally, the in silico approach confirmed the in vitro results. This revealed a strong binding interaction between the plant’s phytochemicals, particularly catechin and myricetin, and the GABA tick protein in a molecular docking study with a docking score of −7.7 and −7.6 kcal/mL, respectively. As a result, this study suggests the use of *P. roxburghii* as a promising anti-tick agent.

**Abstract:**

*Pinus roxburghii*, also known by the name “Himalayan chir pine,” belongs to the Pinaceae family. *Rhipicephalus* (*Boophilus*) *microplus* tick is one of the most significant bovine ectoparasites, making it a major vector of economically important tick-borne diseases. The researchers conducted adult immersion tests (AIT) and larval packet tests (LPT) to investigate the acaricidal effect of *P. roxburghii* plant extract on *R.* (*B.*) *microplus* and its potential modulatory function when used with cypermethrin. Eggs were also assessed for their weight, egg-laying index (IE), hatchability rate, and control rate. After exposure to essential extract concentrations ranging from 2.5 to 40 mg/mL for 48 h, adult female ticks’ oviposition inhibition and unfed *R.* (*B.*) *microplus* larvae’s mortality rates were analyzed. Engorged females exposed to *P. roxburghii* at 40 mg/mL had reduced biological activity (oviposition, IE) compared to positive and negative controls. A concentration of 40 mg/mL of *P. roxburghii* caused 90% mortality in *R.* (*B.*) *microplus* larvae, whereas cypermethrin (the positive control) caused 98.3% mortality in LPT. In AIT, cypermethrin inhibited 81% of oviposition, compared to the 40 mg/mL concentration of *P. roxburghii*, which inhibited 40% of the ticks’ oviposition. Moreover, this study assessed the binding capacity of selected phytocompounds with the targeted protein. Three servers (SWISS-MODEL, RoseTTAFold, and TrRosetta) recreated the target protein RmGABACl’s 3D structure. The modeled 3D structure was validated using the online servers PROCHECK, ERRAT, and Prosa. Molecular docking using Auto Dock VINA predicted the binding mechanisms of 20 drug-like compounds against the target protein. Catechin and myricetin showed significant interactions with active site residues of the target protein, with docking scores of −7.7 kcal/mol and −7.6 kcal/mol, respectively. In conclusion, this study demonstrated the acaricidal activity of *P. roxburghii* extract, suggesting its potential as an alternative natural acaricide for controlling *R.* (*B*.) microplus.

## 1. Introduction

Ticks, which are destructive blood-sucking ectoparasites, cause huge economic losses and food insecurity to both livestock and wildlife [1]. Ticks and tick-borne diseases result in losses of between $13.9 and $18.7 billion per year and a nearly $3 billion deficit in hides and skins for cattle alone [2,3]. These parasites transmit a wide range of protozoan, viral, and bacterial infections that have adverse effects on both livestock and human health. *Rhipicephalus*, *Haemaphysalis*, *Hyalomma*, and *Ornithodoros* are the most common tick genera infesting humans and animals in Pakistan, where they are widespread [4,5]. *Hyalomma* and *Rhipicephalus* species are a significant threat to livestock productivity in Pakistan, and *Rhipicephalus* (*Boophilus*) *microplus*, commonly known as the cow tick, is responsible for transmitting tick fever caused by *Babesia* (*B.*) *bovis*, *B. bigemina*, and *Anaplasma marginale* in this country and globally [6,7].

Parasite resistance has become a significant threat globally, and the use of anti-parasitic drugs by livestock breeders themselves has caused a drug-resistant population, resulting in substantial economic losses in animal productivity [8]. Several genes linked to acaricide resistance have been studied, including the octopamine/tyramine (OCT/Tyr) receptor gene [9] and the C190A alleles of *R.* (*B.*) *microplus* [10]. To address tick resistance, research has led to the development of new management measures, including the selective breeding of tick-resistant cattle, biological control [11,12,13], and the use of entomopathogenic bacteria and fungi [14]. In addition, plant extracts have been studied as an alternative to acaricides for tick populations that are both sensitive [15,16] and resistant [17] to acaricides.

*Pinus roxburghii* Sargent (family: Pinaceae) is a plant with a long history of medicinal use and is commonly known as “chir pine” [18]. *Pinus roxburghii* is a widely distributed tree species found in the Himalayas across Nepal, India, and Pakistan [19]. *P. roxburghii* has a variety of traditional uses [20] and is commonly used in Ayurvedic medicine [21]. It has been used in other parts of its range to treat various conditions, including bronchitis, diaphoresis, ulcers, inflammation, and itching. The present study investigates the potential of *P. roxburghii* as an acaricide against *R.* (*B.*) *microplus* through in vitro and in silico experiments. Previous studies have reported several therapeutic properties of *P. roxburghii*, including antioxidant, antidyslipidemic [20], anti-inflammatory [22], anti-cancer [23], antimicrobial [24], and antidiabetic effects [25], while no acaricidal potential has been reported until now.

The potential use of phytochemicals to target the Gamma-aminobutyric acid (GABA) receptor in ticks, which is essential for their physiological functions, has received growing interest due to its potential to hinder tick nervous system activity, disrupt tick feeding and reproductive processes, and ultimately provide a more sustainable substitute to synthetic acaricides that are becoming more resistant. Hence, additional studies are required to recognize and enhance the utilization of GABA-targeting phytochemicals for tick management [26,27,28].

GABA is a major inhibitory neurotransmitter in vertebrates [29,30]. When released from synaptic vesicles, GABA binds to metabotropic G-protein-coupled receptors or chloride-conducting ligand-gated ion channels [31,32]. GABACls are present in insects and other arthropods [33]. GABACl receptors in insects and arthropods are responsible for muscle relaxation in peripheral neuromuscular locations and inhibitory potentials for neural impulse integration in the central nervous system [34]. Previous studies have demonstrated that the insecticides lindane and cyclodiene [35,36], as well as avermectin and milbemycin [37,38,39], target GABACls. In addition, pesticides such as fipronil, dieldrin, and isoxazoline can antagonize GABA-gated chloride channels in *R.* (*B.*) *microplus* [31]. Dieldrin-resistant mutations in the tick *R.* (*B.*) *microplus* have been reported by Hope, et al. [40]. Furthermore, the GABACls gene has a mutation at codons 868–869 that causes a change from the amino acid Thr to the amino acid Leu.

As the southern cow tick has developed resistance to acaricides, there is a growing need to develop new chemical compounds as part of an integrated tick management strategy. One approach being targeted is the use of natural products, particularly acaricides derived from botanical sources, to counter the increasing frequency of acaricide-resistant tick strains. This study aims to investigate the effectiveness of a crude extract from *P. roxburghii* in controlling *R.* (*B.*) *microplus* at various life cycle stages and to use in silico methods to identify potential inhibitors of RmGABACl.

## 2. Materials and Methods

### 2.1. Collection of Plant Material and Preparation of Extracts

*Pinus roxburghii* leaves used in this study were provided from Mardan, Kyber Pakhtunkhwa, Pakistan (coordinates 34.1986° N, 72.0404° E). The leaves were rinsed with running water and sent to the herbarium at the Department of Botany of the Abdul Wali Khan University Mardan for identification. They were identified as *P. roxburghii* with accession number Awkum.Bot.182.1.2. The leaves were then air-dried for 15 days at room temperature (25 ± 3 °C), followed by grinding using a grinder (YUEYUEHONG Model: HC-3000A, Zhejiang, China) to obtain a thick powder. The powdered leaves were soaked in 96% concentrated ethanol at a ratio of 1:10 (*w*/*v*) to create an extract. Using an orbital shaker incubator (labForce Model 1165U07, Thomas Scientific, Swedesboro, NJ, USA) at 200 rpm, the solution was then shaken. After that, it was filtered three times using Whatman No. 1 filter paper (pore size: 25 µm). The filtered solution was concentrated by evaporation in a rotary evaporator (BUCHI Rotavapor Model: R-300, Flawil, Switzerland) at 40 °C under a vacuum to create a stock solution. Further dilutions were made at concentrations of 2.5, 5, 10, 20, and 40 mg/mL (*w*/*v*, with the weight in mg of stock solution dissolved in volume in mL of distilled water) in 95% ethanol. Once the concentrations were ready, they were stored in the refrigerator at 4 °C until their application, which should not exceed one month. 

### 2.2. Collection and Identification of Rhipicephalus (Boophilus) microplus Ticks

Engorged female ticks were collected from cattle that had not been treated with any type of chemical acaricide in a remote area of Mardan, Pakistan. These ticks were morphologically identified as *R. (B.) microplus* under a stereozoom microscope using standard tick identification keys [41]. Ticks were cleaned and decontaminated by washing them in distilled water containing 1% sodium hypochlorite and drying them with sterile paper towels in the laboratory. Engorged adult female ticks were used for AIT and LPT in this investigation. To encourage egg production, female ticks were incubated at 27 °C and 80% RH. After 10 days of incubation, unfed larvae were removed and placed in plastic syringes.

### 2.3. Adult Immersion Test

The AIT was performed according to standards [42]. First, a total of 210 female ticks were weighed and randomly assigned, with 10 female ticks each, to immerse in one of five concentrations (2.5, 5, 10, 20, and 40 mg/mL) of *P. roxburghii* for 2–3 min. Distilled water was used as a negative control, while 0.6 mL of cypermethrin was used as a positive control. Petri dishes were lined with filter paper to house the 10 ticks. After treatment, ticks in each Petri dish were left at room temperature for 24 h. After 24 h, ticks were moved to muslin-lined glass vials, placed in desiccators, and stored at 28 ± 2 °C with 85 ± 2% relative humidity in an incubator (BioLAB, Model BIBD-101, Toronto, ON, Canada). Tick oviposition and mortality rates were tracked for up to 20 days. The total number of eggs laid by adult ticks exposed to different treatments and tick mortality were recorded. The experiment was carried out in triplicate, and a fresh concentration of the extract was used each time. The same incubation conditions were used, and the hatching rate was determined. The inhibition of oviposition (% IO) was calculated using the given equation:

$$\%IO=\frac{Egg\;laying\;Index\left(control\right)-Egg\;laying\;Index\;(treated)}{Egg\;laying\;Index\;control}\times 100\%$$

The egg-laying index = mean weight of eggs laid ÷ mean weight of engorged females as described by [43].

### 2.4. Larval Packet Test

The LPT was carried out according to standards [42]. A Whatman No. 1 filter paper measuring 3.75 cm by 8.5 cm was covered with 0.6 mL of the extract’s concentration. After 30 min in a 37 °C incubator, the filter paper was to absorb all the phytochemicals. Folding the rectangles in half and sealing them with tape created a pocket that tick larvae could potentially inhabit. Using a paintbrush, approximately 100 larvae (12 days old) were placed in each filter paper pocket. Using adhesive tape, the tops of the packets containing 100 12-day-old larvae were sealed. The packets were placed in an incubator set at 28 ± 1 °C and 85 ± 5% RH. At 24 and 48 h, the packets were opened, and the dead larvae (larvae without light or needle reflexes) were determined. The process was repeated for all the concentrations (2.5 to 40 mg/mL) three times (triplicates), and the same process was performed for the positive control (cypermethrin) and negative control (distilled water) groups using 0.6 mL of ethanol. The number of larvae that died after 24 and 48 h were recorded to determine the lethal concentration (LC) and lethal time (LT). To calculate the fiducial confidence limits (CL) for these values, probit analysis was performed according to Robertson and Liber [44], Finney [45], Wheeler, et al. [46] calculations.

### 2.5. Target Sequence Retrieval

The protein sequence of the *R.* (*B.*) *microplus* GABA-gated chloride channel (RmGABACl) with accession no. KF881797 was obtained from the UniProtKB database in a FASTA file format. The protein’s FASTA sequence was used for further study and model building.

### 2.6. Modeling of the RmGABACl 3D Structure

The revised three-dimensional structure of RmGABACl was determined using two approaches (homology modeling and ab initio design). Homology modeling calculates a protein’s three-dimensional structure using the sequence alignments of homologous proteins as templates. Since the target protein and its template have a high sequence homology rate (>30%), the homology model may be reliable [47]. Ab initio design uses tens of thousands of three-dimensional Protein Data Bank (PDB) models to predict the protein’s structure using machine learning and deep learning. Several studies have used both methods to study protein structure and function [48,49].

### 2.7. Protein Template Search and Homology Modeling

Protein templates were found following recommendations [48]. PSI-BLAST (https://blast.ncbi.nlm.nih.gov/Blast.cgi, accessed on 11 September 2022) and HHpred (https://toolkit.tuebingen.mpg.de/tools/hhpred, accessed on 12 September 2022) were used to compare sequences. The best template was found based on species, sequence coverage/probability, E-value, and sequence identity. Finally, the three-dimensional structure of RmGABACl was designed via the SWISS-MODEL service (https://swissmodel.expasy.org/, accessed on 12 September 2022) using the best template (PDB ID: 6hug.1.B; resolution 3.10 Å) of the PDB database.

### 2.8. Ab Initio 3D Structure Design of RmGABACl

RmGABACl’s three-dimensional structure was predicted using Robetta and TrRosetta from CAMEO (http://cameo3d.org, accessed on 15 September 2022). Using the automated capabilities of the Robetta server, the protein’s structure was predicted and analyzed. Comparative modeling and de novo structure prediction approaches are used to create structural models from input sequences for structure prediction [50]. TrRosetta is a professional server for predicting three-dimensional structures of proteins by using machine learning and deep learning approaches [51].

### 2.9. Model Evaluation of RmGABACl

To ensure the stereochemical stability of the projected models, the PROCHECK module of the PDBSum server 15 was used to verify the percentage of residues in the allowed and favored regions, the glycine and proline residues, the dihedral angle orientations (both phi and psi), and conformations of the backbone [52]. Qualitative evaluation procedures (probable residues residing at a particular distance and observed interactions between model and solvent, i.e., solvation17) were based on ProSA analysis [53]. The values of the ERRAT (non-bounded atoms’ interaction and distribution statistics) score values were used to confirm the product’s quality [54].

### 2.10. Blind Docking Analysis

The blind docking investigation used AutoDock Vina. With its help, how ligands interact with macromolecules may be better understood and predicted. AutoDockTools (ADT), a free graphical user interface (GUI) for the AutoDockVina software (http://vina.scripps.edu/download.html, accessed on 19 September 2022), was used for molecular docking investigations [55]. After conducting an extensive literature review, we have identified specific phytochemicals found in *P. roxburghii* that have previously demonstrated pharmacological, biochemical, and potential biological activity against various parasites and insects. Our selection process for these twenty phytochemicals was based on strong evidence of their bioactivity in previous studies and their chemical structures, which were readily available in the PubChem database. In this study, twenty phytochemicals obtained from *P. roxburghii* were used in docking experiments, which were originally identified and analyzed by *Aditi,* et al. [56] in a related article. The twenty compounds were docked against the RmGABACl protein using AutoDockVina using the default methodology. With an x, y, and z grid point spacing of 0.375 Å, a box with 60 x, 60 y, and 40 z grid points was constructed. The coordinates for the central square on the grid are 30.66 Å, −7.348 Å, and 18.397 Å degrees. The binding energies of nine distinct conformations of each ligand were calculated and ranked using Auto Dock Vina scoring tools. Post-docking studies were performed using the Discovery Studio Visualizer. Target receptor and ligand interactions were examined using the Discovery Studio visualizer by choosing the conformations with the least free binding energy. PDBQT files were generated from processed protein and ligand structures, with rotational torsions for ligands enabled and proteins presumed to be stiff. During docking, a completeness of 2000 was used, and the entire protein was covered by the receptor grid.

### 2.11. Molecular Dynamics Simulation of the Ligand-Receptor Complex

Molecular dynamics simulations are run using the iMOD server. iMODS facilitates the study of such modes by constructing realistic transition paths between two homologous structures [57]. The iMOD server uses normal mode analysis to determine internal coordinates, which in turn enables protein stability assessment (NMA). Methods such as main-chain deformability plots, B-factor values, eigenvalues, covariance matrices, and the elastic network model may be used to demonstrate the protein’s stability.

### 2.12. Statistical Analyzes

All statistical analyses were performed in R version 4.2.0 [58] using the R Studio [59]. A one-way analysis of variance (one-way ANOVA) with post hoc Tukey’s HSD test was performed to statistically evaluate the difference in significance between larval mortality rates at 24 and 48 h and %IO for each concentration using the “agricolae” R package [60]. To determine the lethal concentration (LC) and lethal time (LT) [61] and their corresponding chi-square values for the ethanolic extract, we used probit analysis [45] with a heterogeneity significance (*p*-value) of 0.05 and a fiduciary confidence limit of 95% using the “ecotox” R package [62]. The results were visually displayed using the “ggplot2” R package [63].

## 3. Results

### 3.1. Adult Immersion Test

**Figure 1:**
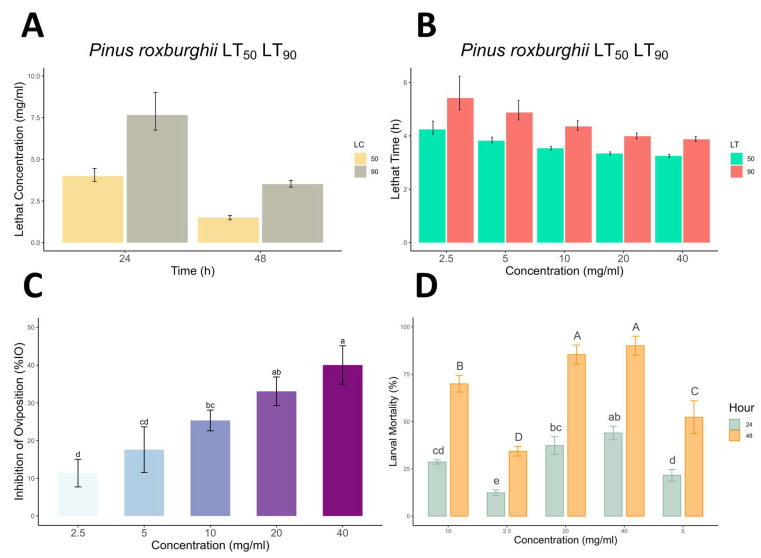
(**A**) The 50% and 90% log transformed lethal concentration values log(LC_50_ and LC_90_) ± its log-transformed confidence limits log(LCL and UCL) of larval mortality; (**B**) The log-transformed lethal time values for 50% and 90% lethality of larvae log(LT_50_ and LT_90_) ± its log-transformed confidence limits log(LCL and UCL), (**C**,**D**) show the one-way ANOVA and post hoc Tukey test showing the significant difference in varying concentrations of %IO and larval mortality, respectively (Bars containing no similar letters are significantly different by Tukey’s HSD test at the 5% level of significance (*p* < 0.05)).

Table 1 and Figure 1C show the percentage of oviposition inhibition for *R.* (*B.*) *microplus* treated with varying doses of the extract. All concentrations of the extract induced a change in egg hatching to some extent in all replicates. The %IO increased with higher concentrations, although these values were not statistically significant compared to the positive control group. The higher concentrations of 20 and 40 mg/mL caused a significant inhibition of oviposition by 33% and 40%, respectively, which is 50% compared to the positive control cypermethrin (81%). Values at lower concentrations were 11%, 17%, and 25% for 2.5, 5, and 10 mg/mL, respectively.

### 3.2. Larval Packet Test

LC_50_, LC_90_, LT_50_, LT_90_, slope, chi-square (X2), intercept, and *p*-values for various concentrations of extracts at varying time intervals were calculated based on the dose-mortality response of collected unfed larvae ticks of *R.* (*B.*) *microplus* using different extracts (Table 2 and Figure 1). The mean larval mortality of *P. roxburghii* at 20 and 40 mg/mL was statistically significant compared to the positive control (cypermethrin) according to the post hoc Tukey’s HSD test, as shown in Table 1 and Figure 1D. The LC_50_ and LC_90_ values were calculated as 4.530 (3.937–5.124) mg/mL and 33.549 (27.881–42.093) mg/mL, respectively, at 48 h intervals, as shown in Table 2. The LT_50_ and LT_90_ values at the highest concentration of 40 mg/mL were 25.82 (24.11–27.38) and 48 (44.18–53.52) h, respectively, as shown in Table 3.

### 3.3. RmGABACl Model Evaluation

The results suggest that PDB ID 6hug.1.B (resolution 3.10 Å), which has the highest sequence identity (42.17%) and species resemblance to RmGABACl, should be further investigated. However, upon comparing the 6hug.1. B and RmGABACl amino acid sequences, it was found that 6hug.1.B was 210 amino acids shorter than RmGABACl. As a result, homology modeling using 6hug.1.B as a template makes it challenging to acquire the whole structure of RmGABACl. Thus, the entire model of RmGABACl was built using ab initio design and then compared to homology models (Figure S2).

**Figure 2:**
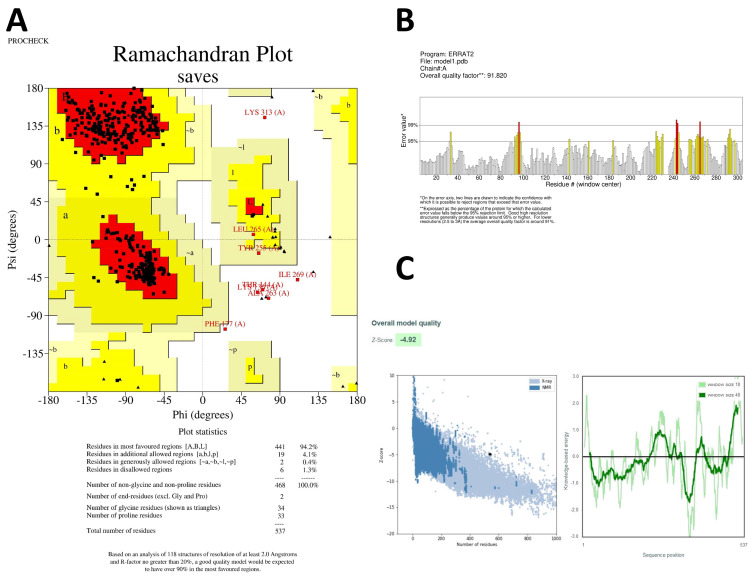
Validation plots and scores for the TrRosetta server’s predicted 3D structure of RmGABACls showing (**A**) the Ramachandran plot where the red, yellow and black colors represents most favorable, favorable, and disallowed region respectively, Phi and Psi bond represent torsion angle which predict the possible conformation of the peptides. (**B**) the ERRAT’s overall quality factor value, and (**C**) the PROSA servers’ Z-score values.

Figure 2 and Supplementary Figures S1 and S2 display the results of the RmGABACl models generated by three servers (SWISS-MODEL, RoseTTAFold, and TrRosetta). The SWISS-MODEL-predicted model had a Prosaweb Z score of −3.27 and an ERRAT value of 77.79, indicating its reliability. The PROCHECK analysis showed that 80.6% of the amino acid residues were in favored regions, 15.5% in allowed regions, 1.9% in generous regions, and 1.9% in disallowed regions, as seen in Supplementary Figure S1. These findings suggest that the model has minimal regional errors, is compatible with the amino acid sequence, and has appropriate stereochemistry for the main chain conformation. The RoseTTAFold and TrRosetta servers used the ab initio design process to create RmGABACl models that included all amino acid residues in relation to the homology model. The model assessment results of the RoseTTAFold server were acceptable. Its projected amino acid sequence structure was shorter than that of RmGABACl, and the model’s ERRAT value was 92.218, with a Prosaweb Z score of −7.06, indicating a reliable model prediction. According to the PROCHECK analysis, 88.7% of the amino acid residues were in the favored region, 8.5% in the allowed region, 1.5% in the generous region, and 1.3% in the disallowed region (Supplementary Figure S2). However, the model built by RoseTTAFold still had accuracy flaws. On the other hand, the model created using the TrRostta server was consistent and reliable, with an ERRAT value of 91.82 and Z-scores from the ProSA web of −4.92. The PROCHECK analysis showed that 94.2% of amino acid residues were in the favored region, 4.1% in the allowed region, 0.4% in the generous region, and 1.3% in the forbidden region (Figure 2). Therefore, the 3D model of RmGABACl built by the TrRosetta server was found to be more reliable and suitable for further investigation.

### 3.4. Molecular Docking Analysis

**Figure 3:**
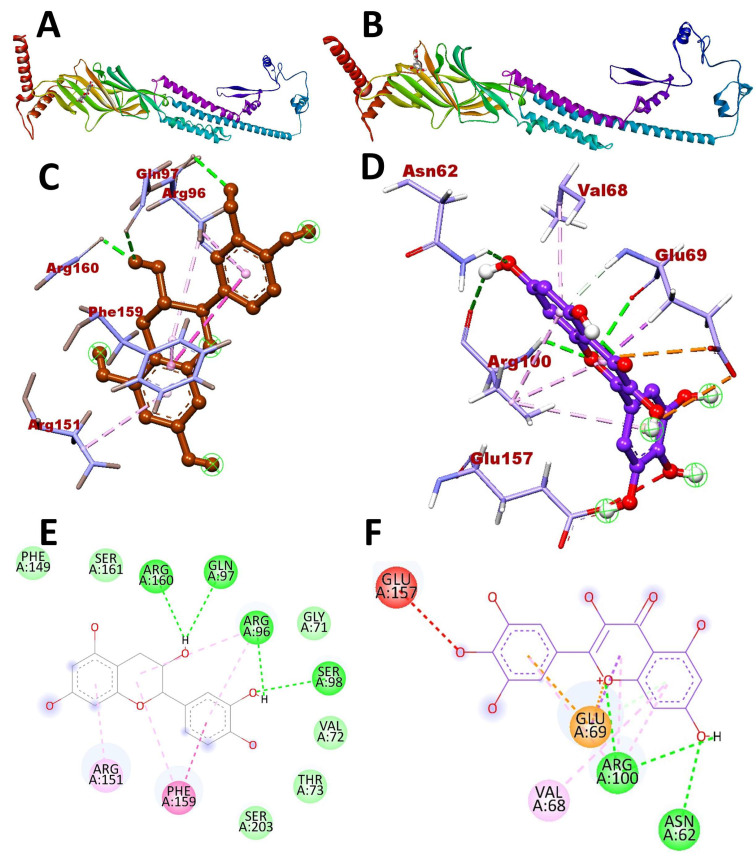
(**A**,**B**) show the complex three-dimensional structures of the RmGABACl proteins; (**C**,**E**) show the highly complex 3D structural interactions of the catechin and RmGABACls and their corresponding 2D interactions; and (**D**,**F**) show the highly complex 3D structural interactions of the myricetin and RmGABACls and their corresponding 2D interactions.

Molecular docking analysis of selected compounds from *Pinus roxburghii* showed a better docking score against *RmGABACl*. Catechin exhibited an exceptional inhibition capacity, with a binding energy value of −7.7 kcal/mol. The hydrogen-bound molecules in the target protein were separated by less than 3.5 Å, indicating a robust hydrogen-bonding connection between the protein and ligands. Catechin revealed four hydrogen bonding interactions with the target receptor, with bond lengths between 2.69 and 1.98 Å, intertwining the amino acid residues of Arg−96, Gln-97, Ser-98, and Arg-160. The amino acid residues of Arg-151, Phe-159, and Arg-96 were in hydrophobic contact. Figure 3C,E depicts the catechin-containing amino acid residue’s hydrogen bonds and hydrophobic interactions with the target protein. Similarly, myricetin exhibited the highest binding affinities (with a binding energy of −7.6 kcal/mL) and displayed two hydrogen bonds (Arg-100 and Asn-62) and lingering hydrophobic interactions with the amino acids Val-68, Glu-69, and Glu-157, as shown in Figure 3D,F. Comparing catechin with other substances, the results demonstrate that it has a better ability to inhibit the target protein of RmGABACl.

### 3.5. Molecular Dynamic Simulation

**Figure 4:**
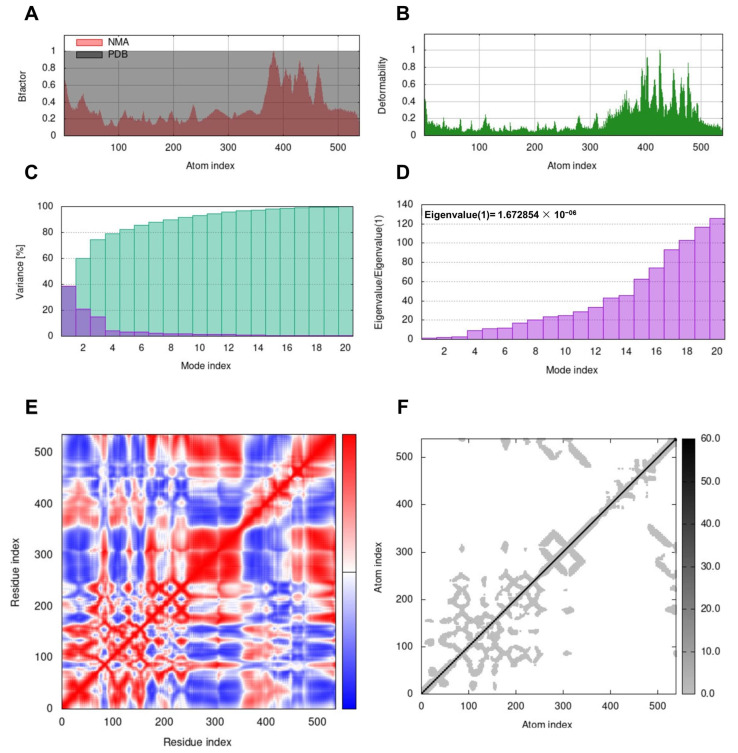
The iMOD server’s normal mode analysis (NMA) outputted the following plots. (**A**) B-factor, (**B**) deformation plot, (**C**) variance plot, (**D**) eigenvalues, (**E**) covariance matrix plot (The anticorrelated, uncorrelated, and correlated states of atomic motion are represented by the blue, white, and red, hues respectively), and (**F**) elastic network model where grey color represents atom connections).

The normal mode analysis (NMA) study, based on internal coordinates, reveals protein mobility. Arrows in the output of the iMOD servers represent domain mobility. The deformability fluctuation map (Figure 4B) represents residues forming a coiled structure, the complex of which is flexible. Consequently, the reduced computed B-factor value of the complex relative to the original B-factor value of the original APB reduced the deformability (Figure 4A). High eigenvalues, a crucial characteristic of a stable structure, are required for a stable complex [64]. The eigenvalue of a modeled docking complex, which is substantially greater for structural stability, is 1.672854 × 10^−6^ (Figure 4D). The covariance matrix is provided in Figure 4E, with the anticorrelated, uncorrelated, and correlated states of atomic motion designated by the hues blue, white, and red, respectively. In a string model, atom connections are represented by elastic springs; however, in a plot matrix (Figure 4F), connections are represented by gray dots.

## 4. Discussion

The need for tick control challenges the sustainability of the dairy sector in areas with favorable conditions for tick growth and spread [65]. Chemical acaricides are commonly used for this purpose, but the development of acaricide resistance in tick species is a major concern, as confirmed by Jyoti, et al. [66]. Furthermore, the use of chemical acaricides can result in environmental pollution and contamination of livestock meat and milk, in addition to promoting tick resistance [67].

In response to these challenges, there is a growing interest in the use of natural plant-based alternatives for tick control. Many researchers have investigated the efficacy of plant extracts and phytochemicals as acaricides, with promising results [68].

Therefore, the present study adds to the existing body of research on the use of natural products for tick control by examining the efficacy of *P. roxburghii* crude extract against *R.* (*B.*) *microplus*. The in silico approach used to identify potential inhibitors of RmGABACl offers a new avenue for the discovery of novel tick control compounds.

The results of this study demonstrate that the ethanolic extract of *P. roxburghii* exhibits high acaricidal efficacy at higher concentrations, effectively killing *R.* (*B.*) *microplus* ticks and larvae, inhibiting oviposition, and hindering egg-laying.

The lethality of the extract was concentration- and time-dependent. The LC_50_ value of *P. roxburghii* in LPT was 4.5303 (3.937–5.124) mg/mL against *R.* (*B.*) *microplus*, which is comparable to the LC_50_ values of 4.06 (3.53–4.58) mg/mL of *Datura innoxia* ethanolic extract [69]. In a related study, the essential oil extracts of *Piper mikanianum* and *P. xylosteoides* showed very good efficacy in killing tick larvae in vitro [70]. Other natural plant-based alternatives, such as *Azadirachta indica* and *Phytolacca dodecandra* leaf extract, have also been reported to have high acaricidal activity against *R.* (*B.*) *microplus*, with the highest mortality rate observed in adult ticks [71]. Chemical acaricides, which are widely used to control ticks, have led to the development of acaricide resistance, environmental pollution, and contamination of livestock products. Therefore, there is a growing interest in plant-based phytochemical alternatives to control ectoparasites, including ticks. This study adds to the growing body of literature on the potential use of natural products, such as *P. roxburghii*, as a promising alternative to synthetic acaricides for tick control.

To advance further in the study, an in silico approach was used to explore the possible binding sites for phytochemicals from *P. roxburghii* with tick protein. Protein receptors or enzymes are the primary targets of acaricides [72]. Thus, twenty different *P. roxburghii* compounds were generated in ChemDraw software and employed as ligands in docking studies against *R.* (*B.*) *microplus’s* gamma-aminobutyric acid gated chloride channel (RmGABACl) neurotransmitter of *R.* (*B.*) *microplus*. The docking scores for catechin and myricetin, with values of −7.7 kcal/mol and −7.6 kcal/mol, respectively, were found to be higher than those of other phytochemicals docked with RmGABACl. This indicates that these two compounds have the potential to act as inhibitors of RmGABACl. The potent binding ability of catechin with target proteins is believed to be attributed to the formation of four hydrogen bonds with protein residues Arg-96, Gln-97, Ser-98, and Arg-160, in addition to numerous hydrophobic interactions. Similarly, myricitin also forms two hydrogen bonds and several hydrophobic bonds with the target protein. To further investigate the ligand–protein interaction, dynamic simulation analysis was conducted, which revealed the contribution of each amino acid residue in the stable structure and low backbone variations of the complex.

We conducted a molecular dynamics simulation of the catechin-RmGABACl complex to investigate its stability and motion. To assess the stability of the ligand–receptor complex, we utilized the iMODS server (http://imods.chaconlab.org, accessed on 1st October 2022), which is based on normal mode analysis (NMA) and predicts the collective movements of macromolecules in internal coordinates, including proteins. The server calculates various parameters of the ligand–receptor complex, such as the elastic network, B-factor, deformability, eigenvalues, variance, and covariance map. The eigenvalue of RmGABACl, which is 1.672854 × 10^−6^, indicates increased stability of the complex. These findings demonstrate the potential of the complex structure as a tick inhibitor. A molecule’s deformability is determined by its ability to change shape at each of its residues, with “highest peaks” being the most common manifestation of high deformability locations.

The structure of the protein data bank (PDB) is utilized for conducting normal mode analysis (NMA) to obtain the B-factor. The NMA mobility is multiplied by a factor of 8pi2 to derive the B-factor. Additionally, the B-factor analysis provides an average root-mean-square (RMS) approximation. The strength of the correlation between the residues in the complex is indicated by the covariance matrix, where a stronger correlation indicates a better complex. Regions with no correlation are represented by the white color, while anticorrelations are indicated by the blue color.

To further elaborate, the PDB is a repository of three-dimensional structures of proteins and nucleic acids that is used as a reference for various structural analyses. NMA is a computational method used to study the collective dynamics of protein molecules by calculating their vibrational modes. The B-factor is a measure of the uncertainty in the positional coordinates of atoms in a protein structure, which can be used to assess the flexibility and mobility of the molecule.

A covariance matrix is a mathematical tool used to determine the correlation between the residues in a protein complex. In the context of protein–protein interactions, a strong correlation indicates that the residues interact with each other and stabilize the complex. On the other hand, an anticorrelation suggests that the residues repel each other and destabilize the complex. The color scheme used to represent the covariance matrix ranges from white for regions with no correlation to blue for regions with anticorrelation.

The red coloration indicates a strong correlation between the residues, and various studies have reported on the diverse activities of catechin and myricitin. Catechin has been reported to exhibit pro-oxidant activity [73], anti-angiogenic activity in endometriosis [74], anti-virulence properties [75], anti-inflammatory effects on human pulp cells [76], and anti-malarial activity [77]. Similarly, myricitin has been found to possess antimicrobial activity against food-borne pathogens [78], antioxidant activity [79], neurobiological activity [80], antidiabetic activity [81], anticancer activity [82], and analgesic activity [83].

It is noteworthy that there have been no previous reports on the acaricidal properties of catechins and myricetin, which makes them novel acaricides that can potentially be used against ticks.

## 5. Conclusions

The acaricidal properties of *P. roxburghii* extract against different life stages of *R.* (*B.*) *microplus* have been demonstrated in vitro, highlighting the potential of these plants for combating other arthropods. However, to determine their impact on host animals, further in vivo studies involving these plants are necessary. These investigations will also help to identify specific components in crude extracts that enhance their acaricidal activity. The study supports the use of *P. roxburghii* leaf extracts as a long-term strategy for integrated tick control due to their anti-ovipositional and other acaricidal activities. This work also involved the molecular docking of twenty phytochemicals from *P. roxburghii* to the RmGABACl protein using Auto Dock VINA software to estimate their binding mechanisms. The results revealed intriguing interactions between catechin and myricetin residues in the active site of the target protein. These findings provide a computational basis for the development of tick inhibitors. However, more research is required to determine the effectiveness of these chemicals as drugs and discover novel ways of controlling livestock ticks.

## Figures and Tables

**Table 1:** The % mean values ± its standard deviation for different concentrations of *Pinus roxburghii* plant leaves extract on *R.* (*B.*) *microplus* larval mortality at 24 and 48 h as well as the inhibition of oviposition in adult female *R.* (*B.*) *microplus*.

Extract	Concentration (mg/mL)	Replicates (*n*)	% Mean ± Standard Deviation		
			% Larval Mortality		% Egg Inhibition
			24 h	48 h	48 h
*P.* *roxburghii*	40	3	44.00 ± 3.605 ^ab^	90.00 ± 5.00 ^a^	40.049 ± 5.050 ^b^
	20	3	37.33 ± 4.726 ^bc^	85.00 ± 5.033 ^ab^	33.079 ± 3.804 ^bc^
	10	3	28.67 ± 1.154 ^cd^	70.00 ± 4.359 ^b^	25.361 ± 2.749 ^cd^
	5	3	21.67 ± 3.055 ^d^	52.00 ± 8.737 ^c^	17.605 ± 6.057 ^de^
	2.5	3	12.33 ± 1.527 ^e^	34.00 ± 2.517 ^d^	11.374 ± 3.657 ^ef^
Control Group	Cypermethrin	3	52.67 ± 4.509 ^a^	98.33 ± 2.887 ^a^	81.240 ± 2.233 ^a^
	Distilled water	3	0.33 ± 0.577 ^f^	1.33 ± 2.309 ^e^	0.401 ± 1.873 ^f^

Means with no similar letters in superscript in the same column are significantly different by Tukey’s HSD test at the 5% level of significance (*p* < 0.05).

**Table 2:** The 50% and 90% lethal concentration values of *Pinus roxburghii* on the in vitro *R.* (*B.*) *microplus* larval mortality at 24 and 48 h.

Time (h)	LC_50_ (mg/mL)	95% Confidence Limits		LC_90_ (mg/mL)	95% Confidence Limits		Slope ± SE	Intercept ± SE	Chi-Square (χ^2^)	*p*-Value
		LCL	UCL		LCL	UCL				
24	54.441	39.524	85.951	2124.390	858.411	8318.730	0.805 ± 0.084	−1.398 ± 0.096	6.446	0.928
48	4.530	3.937	5.124	33.549	27.881	42.093	1.474 ± 0.090	−0.967 ± 0.089	21.457	0.064

LC: Lethal Concentration, LCL: Lower Confidence Limit, UCL: Upper Confidence Limit, SE: Standard Error.

**Table 3:** The lethal time values for 50% and 90% mortality at various concentrations of *Pinus roxburghii* against *R.* (*B.*) *microplus* larvae.

Concentration (mg/mL)	LT_50_ (h)	95% Confidence Limits		LT_90_ (h)	95% Confidence Limits		Slope ± SE	Intercept ± SE	Chi-Square (χ^2^)	*p*-Value
		LCL	UCL		LCL	UCL				
40	25.818	24.110	27.376	48.000	44.184	53.518	4.759 ± 0.407	−6.719 ± 0.607	6.610	0.157
20	28.248	26.503	29.907	53.925	49.226	60.837	4.564 ± 0.383	−6.622 ± 0.579	5.957	0.202
10	34.362	32.089	36.824	77.771	67.053	96.271	3.613 ± 0.358	−5.549 ± 0.552	1.939	0.746
5	45.742	41.539	52.190	131.366	99.621	207.333	2.797 ± 0.361	−4.644 ± 0.561	7.219	0.124
2.5	69.511	58.199	95.103	225.403	145.272	514.087	2.508 ± 0.396	−4.620 ± 0.622	0.993	0.910

LT: Lethal Time, LCL: Lower Confidence Limit, UCL: Upper Confidence Limit, SE: Standard Error.

## Data Availability

Not applicable.

## Supplementary Materials

The following supporting information can be downloaded at: https://www.mdpi.com/article/10.3390/ani13081388/s1, Figure S1: Validation plots and scores for the SWISS-MODEL’s predicted 3D structure of RmGABACls showing A. Ramachandran Plot, B. the ERRAT’s overall quality factor value, and C. The PROSA servers’ Z-score values;, Figure S2: Validation plots and scores for the Robetta servers’ predicted 3D structure of RmGABACls showing A. Ramachandran Plot, B. the ERRAT’s overall quality factor value, and C. The PROSA servers’ Z-score values.
