# Supplementary material for: In Vitro and In Silico Protocols for the Assessment of Anti-Tick Compounds from Pinus roxburghii against Rhipicephalus (Boophilus) microplus Ticks
Source: Animals (Basel). 2023 Apr 18;13(8):1388. doi: 10.3390/ani13081388 (PMC10135231; doi:10.3390/ani13081388)
Supplement: Supplementary file 1 [file animals-13-01388-s001.zip › animals-2121473-supplementary.pdf]

Supplementary Figures

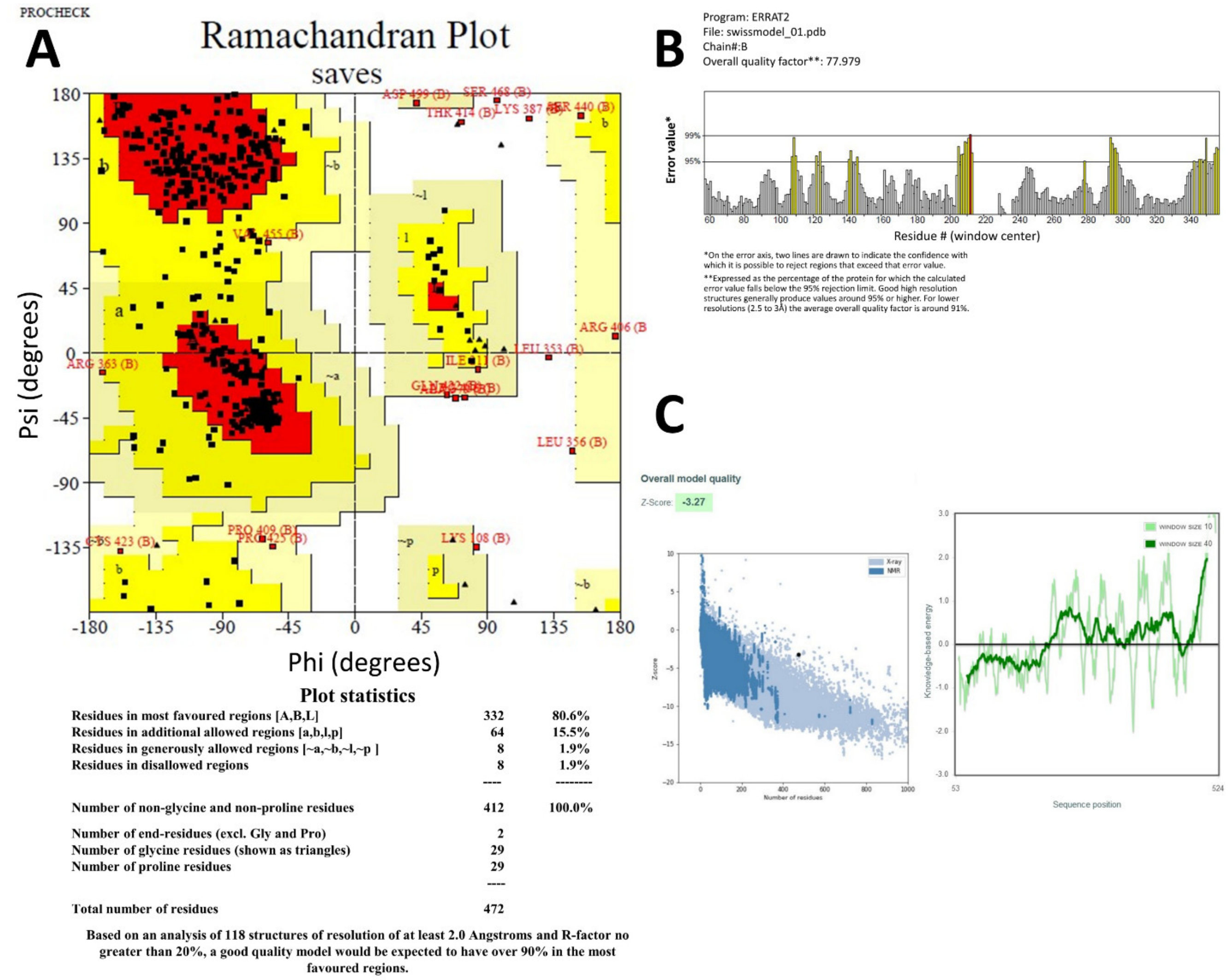

Supplementary Figure S1. Validation plots and scores for the SWISS-MODEL's predicted 3D structure of RmGABACls showing **A.** Ramachandran Plot, **B.** the ERRAT's overall quality factor value and **C.** The PROSA servers's Z-score values.

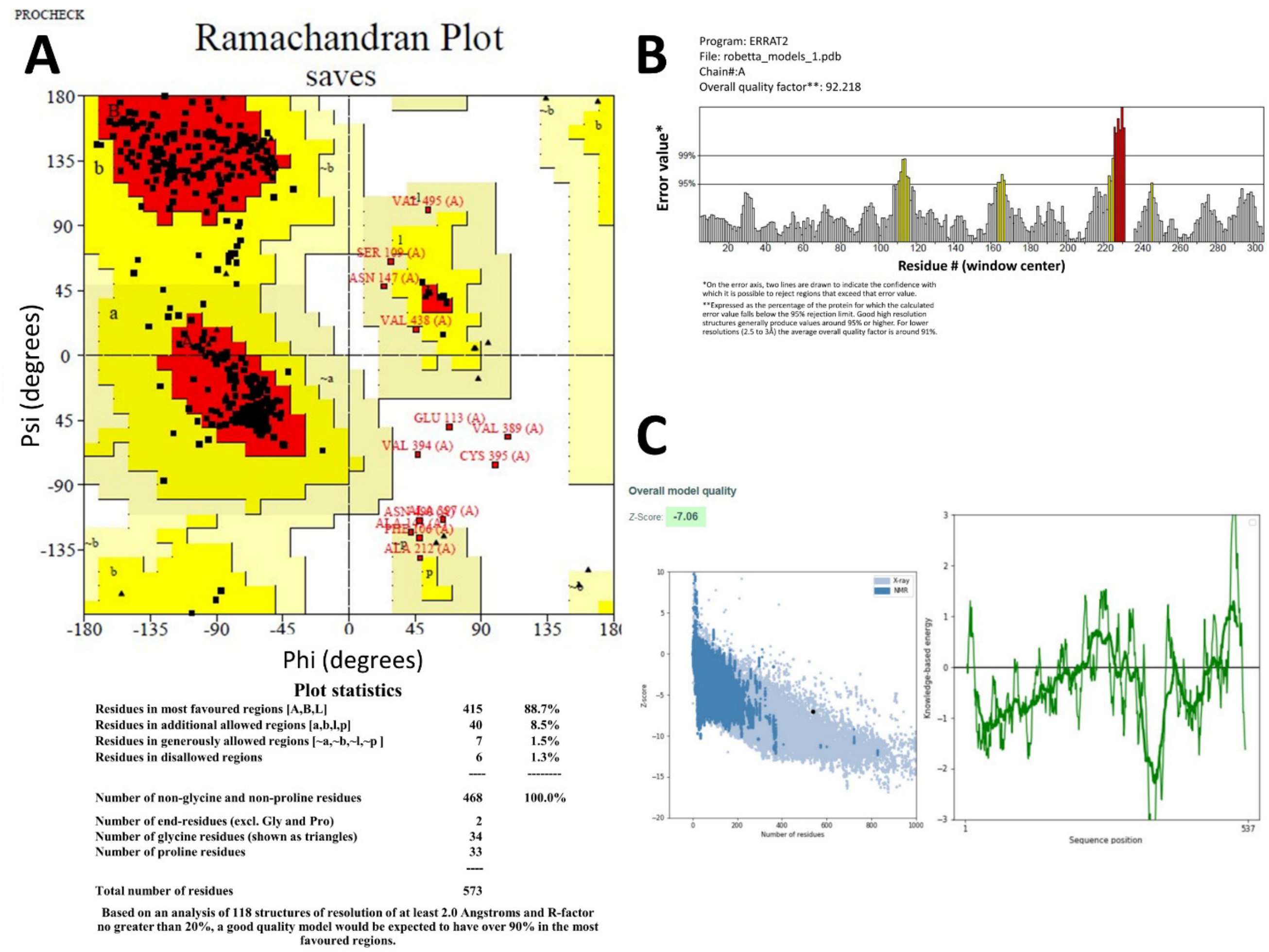

Supplementary Figure S2. Validation plots and scores for the Robetta server's predicted 3D structure of RmGABACls showing **A.** Ramachandran Plot, **B.** the ERRAT's overall quality factor value and **C.** The PROSA servers's Z-score values.
